# Age, sex and physical activity differences in the knee joint loadings during walking—a cross-sectional study

**DOI:** 10.3389/fbioe.2026.1850746

**Published:** 2026-06-24

**Authors:** Hanna Zadoń, Katarzyna Nowakowska-Lipiec, Piotr Szaflik, Steriani Elavsky, Jaroslav Uchytil, Petr Kutac, Jiri Skypala, Daniel Jandacka

**Affiliations:** 1 Independent Researcher, Zabrze, Poland; 2 Department of Biomechatronics, Faculty of Biomedical Engineering, Silesian University of Technology, Zabrze, Poland; 3 Human Motion Diagnostic Center, Department of Human Movement Studies, University of Ostrava, Ostrava, Czechia

**Keywords:** age differences, gait analysis, knee joint forces, physical activity, runners, sex differences, walking analysis

## Abstract

**Introduction:**

The aim of this cross-sectional cohort study was to assess differences in internal knee joint loading during walking in relation to age, sex, and physical activity level.

**Methods:**

Kinematic and dynamic data obtained from experimental investigations were analysed and used to develop a mathematical model of walking within the AnyBody Modeling System. Statistical Parametric Mapping (SPM) was used to assess the associations between individual parameters and the resultant knee joint reaction force.

**Results:**

The results demonstrated that age-, sex-, and physical activity-related differences in knee joint loading varied across phases of the walking gait cycle. A statistically significant association with physical activity (p < 0.05) was observed in the youngest group of women (18–25 years) and in the 46–65-year age groups in both sexes, with physically inactive individuals exhibiting higher force values during the mid-phase of the walking gait cycle (30%–50%). Sex-related differences were equivocal, with higher resultant knee joint reaction force in men identified only in selected age groups (18–25 and 56–65 years) and during the early stance phase (10%–22%). Comparison between younger and older groups revealed an age-related shift in peak loading towards the early phase of foot-ground contact, which may reflect compensatory changes in locomotor strategy.

**Discussion:**

The findings indicate that age, sex, and physical activity are significant factors that should be taken into account in the analysis of knee joint biomechanics, both in the context of preventing degenerative changes and in the planning of individualised training programmes.

## 1 Introduction

Age and physical activity level are major determinants of musculoskeletal function, influencing movement patterns and joint loading during locomotion. Biological processes associated with ageing lead to gradual structural and functional changes within the locomotor system, including, among others, reductions in muscle mass and strength (Boyer et al., 2012; Núñez-Lisboa and Dewolf, 2025), decreased joint stability (Peitola et al., 2025) and modifications of movement patterns (Begg and Sparrow, 2006; Favre et al., 2014). These changes may substantially affect the transmission and distribution of mechanical loads within the locomotor system (Nowakowska-Lipiec et al., 2025).

Regular physical activity may constitute an important factor modifying changes occurring within the musculoskeletal system. Numerous scientific reports confirm its beneficial effect on the musculoskeletal function, particularly with respect to maintaining muscle mass and strength, an increase in bone mineral density (Kohrt et al., 2004) and a reduced risk of developing degenerative joint diseases (Kraus et al., 2019). Regular physical activity also promotes improvements in joint stability and neuromuscular control, which may lead to more effective movement control (Núñez-Lisboa and Dewolf, 2025). Regardless of exercise types, regular sport activity has a positive impact on health, makes it possible to maintain the function of lower limbs with age, contributing to healthy movement patterns (Borgia et al., 2022).

It should be emphasised, however, that the musculoskeletal system response to physical effort may differ between sexes, reflecting anatomical differences, body proportions and physiological adaptations of women and men (Chehab et al., 2017). Consequently, age, sex and physical activity may contribute to variability in locomotor system.

Age-, sex-, and activity-related differences in musculoskeletal system function, including muscle strength, neuromuscular control, and walking pattern, may influence the magnitude and distribution of internal loads transmitted through the joints during daily activities, such as gait (Nowakowska-Lipiec et al., 2023; Zadoń et al., 2023). The knee joint, as one of the primary joints responsible for load transmission during locomotion, is particularly exposed to internal forces arising from both body mass and lower limb muscle activity (Zadoń et al., 2024). Prolonged exposure to elevated internal loading may be associated with increased mechanical stress on joint structures and has been suggested as a factor related to the development of degenerative changes (Baliunas et al., 2002).

Despite numerous studies analysing the effects of age, sex, or physical activity level on gait biomechanics (DeVita and Hortobagyi, 2000; Begg and Sparrow, 2006; Boyer et al., 2012; Schloemer et al., 2017; Obrębska et al., 2020), the above-mentioned factors have not been jointly examined in the context of internal knee joint loading during walking. Internal knee joint loading reflects the forces transmitted within the joint as a result of body mass, ground reaction forces, and muscle action during movement. Unlike external joint moments, which are commonly used as indirect indicators of loading (Hafer et al., 2019; Borgia et al., 2022), internal joint reaction forces are estimated using musculoskeletal models based on kinematic and kinetic gait data. Therefore, they provide a more direct, although model-dependent, representation of loading within the joint. However, relatively few studies have addressed the issue of internal forces (Kutzner et al., 2010; Obrębska et al., 2020), particularly in the context of age-, sex-, and physical activity-related differences within a single cohort. Consequently, the extent to which these factors are associated with knee joint loading patterns during walking remains insufficiently understood.

The aim of this cross-sectional cohort study was to assess differences in internal knee joint loading during walking, expressed as the model-derived resultant knee joint reaction force, according to age, sex, and physical activity level using musculoskeletal modelling. The following research hypotheses were formulated.

1. Older adults are characterised by lower internal knee joint loading during walking compared with younger individuals;
2. Men exhibit higher knee joint loading than women;
3. Regular physical activity is associated with lower internal knee joint loading during walking.

## 2 Materials and methods

Based on experimentally obtained kinematic data and ground reaction forces, knee joint loading was estimated using a mathematical model developed within the AnyBody Modeling System.

### 2.1 Ethics statement

The experimental study was conducted according to the guidelines of the Declaration of Helsinki, and approved by the Ethics Committee of University of Ostrava, Czech Republic (protocol code OU-87674/90-2018 and date of approval 29 November 2018).

### 2.2 Participants

Sample size estimation was performed using G*Power 3.1 software, assuming a significance level of α = 0.05, statistical power (1−β) = 0.80 and an effect size of ES = 0.25. The estimation was based on parameters reported in Obrębska et al. (2020). The analysis revealed that a minimum sample size of 200 participants was required. As a result, a total of 200 individuals were included in the study – 100 women and 100 men–belonging to the following age groups: 18–25, 26–35, 36–45, 46–55 and 56–65. The individuals subjected to the study included 100 active runners (active runners) and 100 physically inactive individuals (inactive controls). Each age × sex × physical activity subgroup consisted of 10 participants, resulting in a balanced study design across all analyzed factors (Table 1). Physically active and inactive participants were pair-matched within each age and sex group based on age, BMI, and self-selected walking speed to obtain comparable groups.

**TABLE 1:** Characteristics of participants taking part in walking analysis.

Sex	Age group	N	Activity level	RUNHIS	Age [years]	Body mass [kg]	Body height [cm]	BMI [kg/m^2^]	Walking speed [km/h]
Female	18-25	10	Inactive	1 ± 0.0	21.1 ± 2.1	59.7 ± 6.3	163.4 ± 5.6	22.3 ± 1.3	4.2 ± 0.2
		10	Active	3.2 ± 0.4	20.9 ± 2.3	65.2 ± 4.4	170.9 ± 5.5	22.3 ± 1.2	4.3 ± 0.2
	26-35	10	Inactive	1 ± 0.0	30.3 ± 3.0	63.5 ± 5.8	167.7 ± 4.5	22.6 ± 1.6	4.3 ± 0.2
		10	Active	3.4 ± 0.5	30.3 ± 3.2	61.8 ± 5.1	165.7 ± 3.4	22.5 ± 1.7	4.5 ± 0.3
	36-45	10	Inactive	1 ± 0.0	41.7 ± 2.3	60.6 ± 5.8	164.8 ± 5.9	22.3 ± 1.3	4.5 ± 0.3
		10	Active	3.2 ± 0.4	41.5 ± 2.4	64.7 ± 6.7	170.2 ± 5.8	22.3 ± 1.5	4.6 ± 0.2
	46-55	10	Inactive	1 ± 0.0	50.0 ± 2.9	62.0 ± 6.7	165.6 ± 6.7	22.6 ± 1.6	4.4 ± 0.2
		10	Active	3.1 ± 0.3	49.5 ± 2.9	62.8 ± 4.2	167.2 ± 4.4	22.5 ± 1.7	4.5 ± 0.2
	56-65	10	Inactive	1 ± 0.0	58.5 ± 1.4	64.9 ± 7.6	167.8 ± 6.3	23.0 ± 2.4	4.5 ± 0.3
		10	Active	2.7 ± 0.8	57.6 ± 2.0	64.0 ± 8.1	166.9 ± 5.8	23.0 ± 3.0	4.6 ± 0.5
Male	18-25	10	Inactive	1.2 ± 0.6	20.4 ± 1.9	74.4 ± 9.8	180.2 ± 5.0	22.8 ± 2.2	4.4 ± 0.2
		10	Active	3.4 ± 0.5	20.4 ± 1.4	73.1 ± 7.7	180.2 ± 4.5	22.5 ± 2.0	4.3 ± 0.2
	26-35	10	Inactive	1 ± 0.0	30.9 ± 2.8	74.2 ± 6.2	178.8 ± 5.5	23.2 ± 0.9	4.3 ± 0.2
		10	Active	3.3 ± 0.5	30.9 ± 2.7	76.2 ± 6.1	181.9 ± 5.2	23.0 ± 1.2	4.5 ± 0.3
	36-45	10	Inactive	1 ± 0.0	41.2 ± 2.5	80.8 ± 7.7	183.3 ± 5.8	24.0 ± 1.4	4.5 ± 0.3
		10	Active	3.2 ± 0.4	40.7 ± 2.6	80.0 ± 5.2	182.7 ± 6.7	24.0 ± 1.3	4.5 ± 0.2
	46-55	10	Inactive	1 ± 0.0	49.4 ± 3.0	80.5 ± 9.6	180.0 ± 6.7	24.8 ± 2.0	4.6 ± 0.3
		10	Active	3.5 ± 0.5	49.8 ± 3.1	76.2 ± 6.2	175.8 ± 5.6	24.7 ± 1.8	4.5 ± 0.2
	56-65	10	Inactive	1 ± 0.0	60.6 ± 2.9	82.5 ± 8.8	179.4 ± 5.4	25.6 ± 2.0	4.5 ± 0.3
		10	Active	2.7 ± 0.7	60.9 ± 2.5	82.4 ± 10.2	177.7 ± 7.0	26.0 ± 2.0	4.6 ± 0.3

RUNHIS (Running History) – variable describing participants’ self-reported weekly running distance, assessed by selecting one of seven categories: (1) 0–5 km, (2) 6–10 km, (3) 11–20 km, (4) 21–30 km, (5) 31–40 km, (6) 41–50 km, (7) ≥51 km per week.

The focus on runners was motivated by the need to include a group characterized by a consistent and relatively homogeneous pattern of regular physical activity with well-defined loading characteristics of the musculoskeletal system. Physically inactive participants were defined as those who were physically capable of running but did not engage in this activity and did not meet public health recommendations for minimum levels of physical activity. In turn, active runners were required to engage in at least 150 min per week of moderate physical activity or 75 min per week of intense physical activity (or an equivalent combination of both), including running. In addition, participants were required to run regularly for at least 6 weeks, covering a minimum weekly distance of 10 km, and to declare their intention to continue this activity over the subsequent 12 months. Weekly running distance was assessed using a self-reported Running History (RUNHIS) variable, which enabled a standardized classification of participants, according to weekly running volume, into one of seven predefined categories: categories: (1) 0–5 km, (2) 6–10 km, (3) 11–20 km, (4) 21–30 km, (5) 31–40 km, (6) 41–50 km, or (7) ≥51 km per week. Although the RUNHIS classification was based on self-reported data, it allowed for consistent categorization of regular running activity across participants.

Exclusion criteria included smoking, the presence of acute health conditions preventing physical activity within the preceding 6 weeks (e.g., surgery, pain or injury) as well as other acute illnesses. Because of the specific nature of the project, additional exclusion criteria included contraindications to magnetic resonance imaging (MRI) or dual-energy X-ray absorptiometry (DXA). A detailed description of the participant recruitment process is provided in the publication by Jandačka et al. (Jandacka et al., 2020), whereas the characteristics of the study groups are presented in Table 1.

### 2.3 Experimental data

Lower limb kinematic data during walking were recorded using a Motion Capture System (Qualisys, Göteborg, Sweden) equipped with 10 motion capture cameras. Ground reaction force data were collected using three force platforms (Kistler Instruments AG, Winterthur, Switzerland) embedded in the measurement walkway. A total of 48 passive markers were placed on the lower limbs and pelvis of each participant, at specifically defined anthropometric points, in accordance with the methodology described in a publication by Malus et al. (2021). Marker placement included anatomical landmarks and marker cluster plates were attached to the thigh and shank segments. Before each measurement session, the global coordinate system was calibrated using a wand calibration kit according to the manufacturer’s recommendations (Qualisys, Sweden). Kinematic data and ground reaction forces were recorded at sampling frequencies of 240 Hz (Motion Capture cameras) and 2,160 Hz (Kistler force platforms), respectively. Experimental tests started with the recording of a static standing posture, followed by walking data collection. Participants were tasked with walking naturally along the measurement walkway in accordance with the designated direction, maintaining a free walking rate and with minimal interference with its natural pattern. For each participant, walking measurements were performed six times. Trials with complete marker trajectories and correct force platform contacts were selected for further analysis. Marker trajectories and ground reaction force data were labelled and processed using Qualisys Track Manager software and subsequently exported as C3D files, which constituted the input data for musculoskeletal simulations in the AnyBody Modeling System.

### 2.4 Musculoskeletal modelling and simulation

The simulations involved the use of a musculoskeletal model available in the repository of the AnyBody Modeling System, version 8.0 (AnyBody Technology, Aalborg, Denmark) (Damsgaard et al., 2006). The model was constructed from rigid bodies representing individual body segments, connected by kinematic pairs with degrees of freedom corresponding to the mobility of a given joint (Damsgaard et al., 2006). The muscular system was modelled using linear elements corresponding to muscle actons. In this study, the AnyMuscleModel muscle model was applied, where a constant muscle force independent of muscle work conditions was assumed. The model adopted rigid-body mechanics and did not incorporate subject-specific bone geometry, cartilage properties, or muscle architecture. Muscle forces and joint reaction forces were estimated indirectly, using inverse dynamics and static optimization, rather than measured experimentally.

**FIGURE 1:**
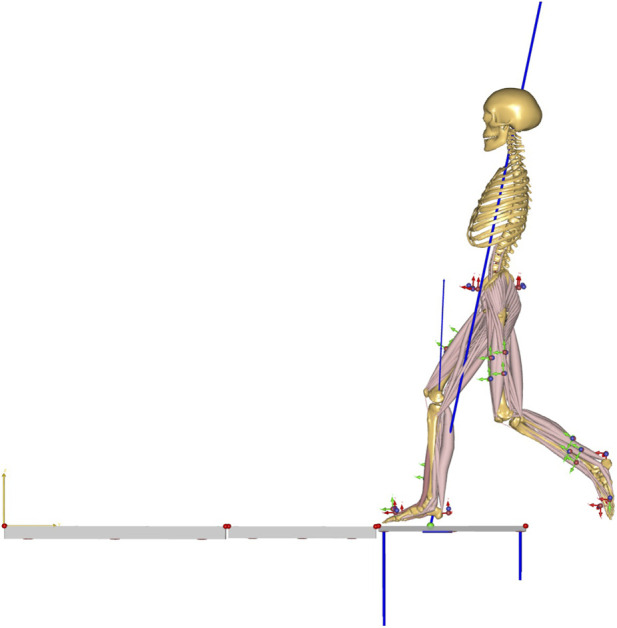
Example of walk simulation in the AnyBody Modeling System.

In addition, the Scaling Length Mass Fat scaling method (available in the Anybody software repository) was applied in the model. Based on information on the body height, body mass and percentage fat tissue content (estimated on the basis of the body mass index, BMI), this method enabled scaling of body segment dimensions and related muscle forces for each individual. On the basis of kinematic and dynamic data recorded during the experimental trials, a mathematical walking model was developed within the Any Body Modeling System (AnyBody Technology). The simulations involved two stages: (1) static simulations of the standing posture and (2) dynamic simulations of walking. The static standing posture simulations enabled identification of model parameters, i.e., the alignment of marker positions initialised in the model with the marker positions recorded during the experimental trials. These data were subsequently used to perform the dynamic simulations of walking. Both the static standing posture and walking simulations were performed using a full-body human musculoskeletal model (GaitFullModel; Figure 1). However, owing to the lack of upper limb kinematic data, the upper limbs were excluded from the modelling. Standing posture and walking were reconstructed based on the motion trajectories of 32 markers and the recorded ground reaction force data. In total, 1,200 walk simulations were performed, corresponding to six walking gait cycles analysed for each study participant.

The identification of loads occurring in the musculoskeletal system in the AnyBody was based on solving an inverse dynamics problem, followed by the application of static optimization. The adopted optimisation criterion was the movement control criterion, assuming the minimization of the cubic sum of the ratio of muscular force to maximum force (the default criterion of polynomial optimisation) (Damsgaard et al., 2006).

The conducted numerical simulations enabled the quantification of resultant knee joint reaction forces during walking, defined as the vector sum of the compressive, antero-posterior shear, and medio-lateral shear components acting on the knee joint at each instant. For further analyses, loading values were normalised in relation to the body weight (BW).

**FIGURE 2:**
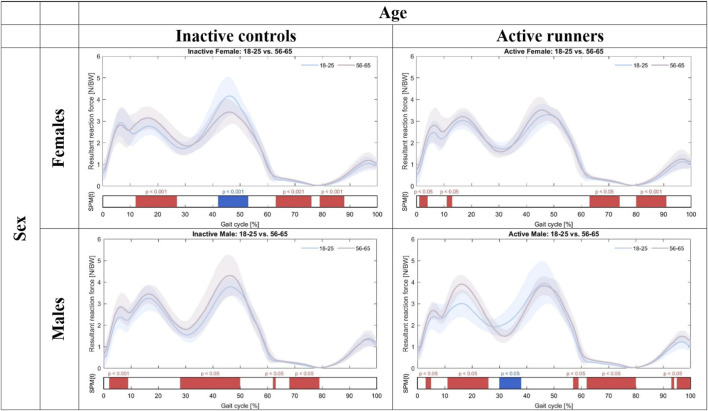
Resultant knee joint reaction forces during walking according to age (18–25 vs. 56–65 years). Solid lines represent mean values, while shaded areas indicate standard deviation. The bar beneath the plot presents the results of the statistical parametric mapping analysis (SPM{t}). Blue and red segments of the bar indicate walking gait cycle intervals in which statistically significant differences between groups were observed (p ≤ 0.05); blue indicates higher loading in the 18–25-year group, whereas red denotes higher loading in the 56–65-year group.

For additional discrete-point analysis, characteristic points of the curve of BW-normalized resultant knee joint reaction forces were identified during the stance phase (Figure 2). P1 was defined as the first local maximum occurring in the early stance phase, typically between 10% and 20% of the gait cycle. P2 was defined as the local minimum between P1 and P3, corresponding to the mid-stance phase. P3 was defined as the subsequent local maximum occurring in the late stance phase, typically between 35% and 55% of the gait cycle. This approach enabled consistent identification of P1, P2, and P3 across participants despite individual variability in the shape of the loading curve. This definition was applied consistently to both two- and three-peak loading patterns, with P1–P3 selected according to their temporal location within the stance phase.

### 2.5 Statistical analysis

Statistical analyses of the resultant knee joint reaction force courses during walking were performed using one-dimensional Statistical Parametric Mapping (SPM) (version M.0.4.5) (Pataky, 2010) adopting significance level p ≤ 0.05. The SPM analysis was performed across the entire normalised gait cycle (0%–100%) to identify time intervals characterised by statistically significant between-group differences. The critical threshold for statistical significance was determined automatically by random field theory implemented in the SPM framework. Comparisons of resultant knee joint reaction forces between groups, differing in a physical activity level, age and sex, were conducted using Unpaired t-tests. In addition, the size of effect was determined using Cohen’s d coefficient (Cohen, 1988).

For discrete-point analyses, the values of the BW-normalized resultant knee joint reaction force at the characteristic points P1, P2, and P3 were analysed. Normality of distribution was assessed using the Shapiro–Wilk test. Depending on data distribution, between-group comparisons were performed using either independent-samples Student’s t-tests or Mann–Whitney U tests. Effect size was calculated using Cohen’s d coefficient (Cohen, 1988) for the Student’s t-test and the rank biserial correlation for the Mann–Whitney U test.

Calculations were performed using MATLAB R2025a (MathWorks, Natick, MA, United States), Statistica 14.0.1.25 software (TIBCO Inc., Palo Alto, CA, United States), and JASP 0.18.3.0 (Jeffreys’s Amazing Statistics Program, University of Amsterdam, Amsterdam, Netherlands).

## 3 Results

This section presents the results of the walk simulations performed using the AnyBody Modeling System. The resultant knee joint reaction forces were normalised in relation to body weight and presented as courses within the full walking gait cycle (0%–100%).

Figure 2 shows differences in the resultant knee joint reaction force between the youngest and oldest age groups (18–25 vs. 56–65 years). Figure 4 presents sex-related differences between women and men, whereas Figure 5 shows differences between physically active and inactive participants. To compare the time courses, statistical parametric mapping analysis (SPM{t}) was applied. Statistically significant intervals, identified using SPM{t}, are indicated in the corresponding figures by colour-coded bars displayed beneath each diagram, whereas mean Cohen’s d values for these intervals are reported in Supplements 1–3. The colour coding of the bars reflects the direction of the observed between-group differences.

**FIGURE 3:**
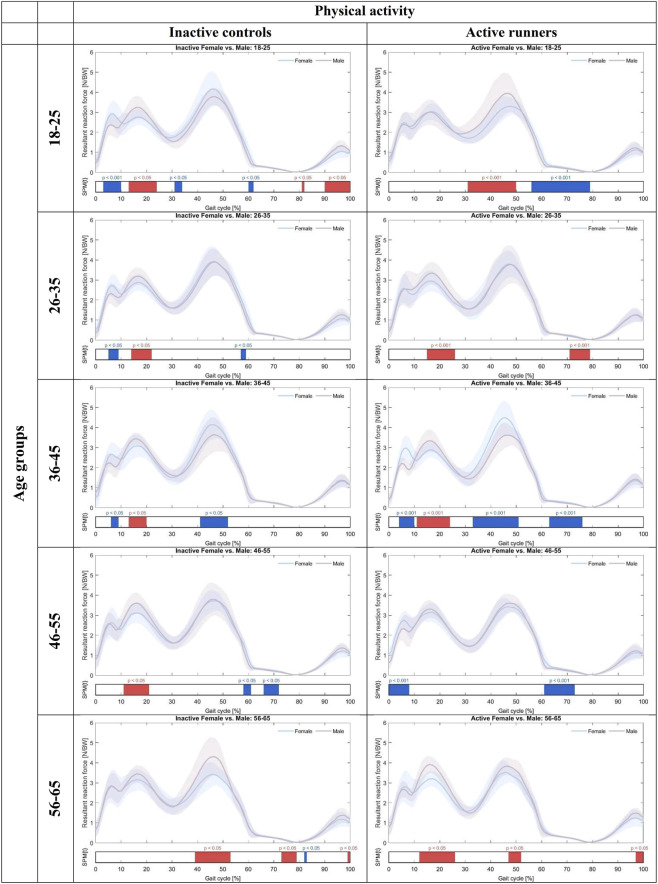
Resultant knee joint reaction forces during walking according to sex (women vs. men). Solid lines represent mean values, whereas shaded areas indicate standard deviation. The bar beneath the diagram indicates walking gait cycle intervals in which statistically significant differences were identified (SPM{t}, p ≤ 0.05); red indicates higher loading in men, whereas blue indicates higher loading in women.

In physically inactive women, older participants (56–65 years) demonstrated significantly higher BW-normalized resultant knee joint reaction force during the early stance phase, whereas younger women (18–25 years) showed higher resultant knee joint reaction force during the late stance phase (Figure 3). In physically active women, age-related differences were less pronounced and were mainly observed in the terminal part of the gait cycle. In physically inactive men, significant age-related differences in BW-normalized knee joint loading were observed during the early and mid-stance phases, as well as at the beginning of the swing phase. In physically active men, significant age-related differences were also identified, with higher loading in older men observed during the first part of the stance phase and during the swing phase.

**FIGURE 4:**
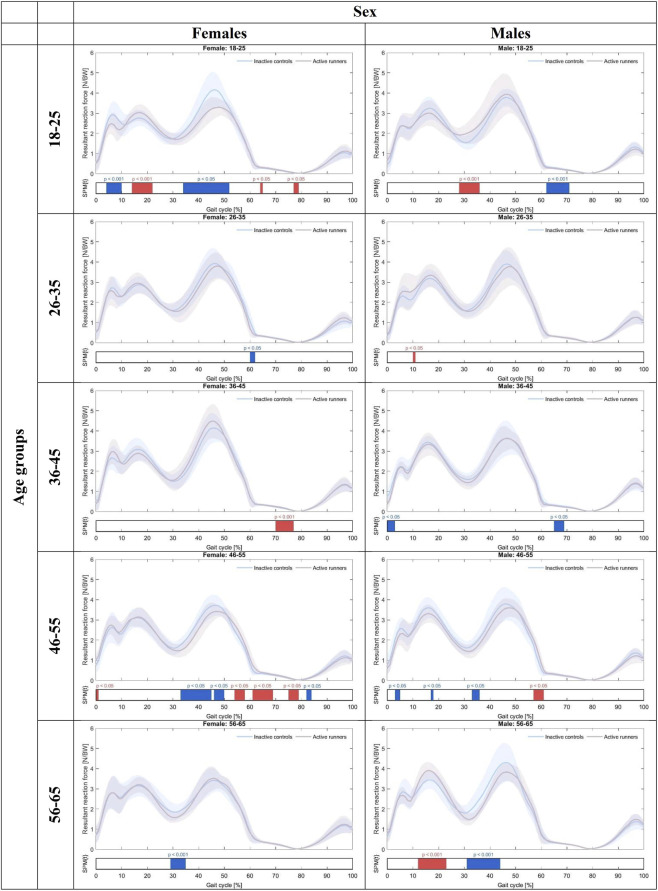
Resultant knee joint reaction forces during walking according to physical activity level (physically active vs. physically inactive individuals). Solid lines represent mean values, whereas shaded areas indicate standard deviations. The bar beneath the diagram indicates walking gait cycle intervals in which statistically significant differences were identified (SPM{t}, p ≤ 0.05); blue indicates higher loading in physically inactive individuals, whereas red indicates higher loading in physically active individuals.

Higher BW-normalized resultant knee joint reaction force in men was observed primarily in the 56–65-year age group in both physically active and inactive participants, as well as in physically active individuals aged 18–25 years (Figure 4). In contrast, women aged 36–45 years exhibited significantly higher BW-normalized resultant knee joint reaction force than men regardless of physical activity level.

**FIGURE 5:**
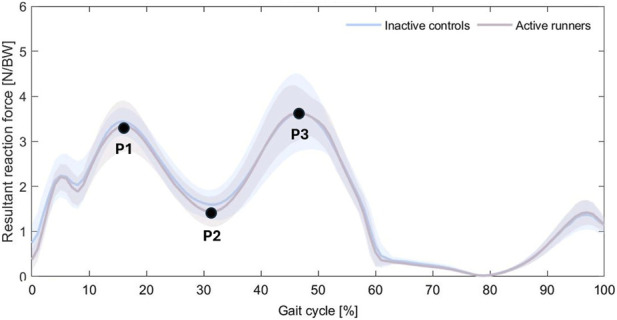
Example time course of the resultant knee joint reaction force during the walking gait cycle. P1 denotes the first peak of knee joint loading occurring in the early stance phase (≈10–20% of the walking gait cycle), P2 represents the local minimum in the mid-stance phase, and P3 denotes the second peak occurring in the late stance phase (≈35–55% of the walking gait cycle).

Among participants aged 26–45 years, no extended gait-cycle intervals with significant differences in BW-normalized resultant knee joint reaction force were observed between inactive individuals and runners (Figure 5). In contrast, runners demonstrated significantly lower BW-normalized resultant knee joint reaction force than inactive participants, namely, in women aged 18–25 and 46–55 years, as well as in men aged 56–65 years, particularly between approximately 30% and 50% of the gait cycle. This interval corresponds to the stance phase, during which the resultant knee joint reaction force typically reaches its highest values. The resultant knee joint force had a characteristic course (Figure 5) — there were two or three characteristic peaks during the stance phase, with a maximum always in the early stance phase–indicated as P1 (usually between 10% and 20% of the walking gait cycle) and a subsequent maximum in the late stance phase–indicated as P3 (between 35% and 55% of the walking gait cycle). Between these two maxima, i.e., P1 and P3, the course of the resultant knee joint reaction force had one local minimum in the middle of the stance phase (indicated as P2). Table 2 presents the mean values of resultant knee joint reaction force at points P1, P2 and P3, along with the corresponding percentages of the walking gait cycle at which they occurred. Statistically significant differences between the inactive group and runner group were observed at selected points, where the magnitude of these differences was quantified using effect size measures (ES).

**TABLE 2:** Mean resultant knee joint reaction force at characteristic stance-phase points (P1–P3) and between-group differences (Inactive vs. Runners).

Sex	Age group	Activity level	P1 [N/BW] mean ± SD	% Cycle P1 mean ± SD	P2 [N/BW] mean ± SD	% Cycle P3 mean ± SD	P3 [N/BW] mean ± SD	% Cycle P3 mean ± SD
Females	18-25	Inactive	3.3 ± 0.5	11.2 ± 5	1.6 ± 0.2	30.2 ± 3.6	4.3 ± 0.8	46.9 ± 2.6
		Active	3.2 ± 0.4	14.9 ± 4.2	1.6 ± 0.2	31.7 ± 3.9	3.5 ± 0.5	48.1 ± 2.6
		p-value (ES)	0.269 (0.117)	< 0.001*(-0.38)	0.985 (-0.003)	0.029* (-0.404)	< 0.001* (0.624)	0.007* (-0.281)
	26-35	Inactive	3.1 ± 0.5	13.1 ± 4.9	1.5 ± 0.2	30.3 ± 2.5	4.1 ± 0.7	47.1 ± 2.5
		Runner	3.3 ± 0.5	12.3 ± 4.9	1.4 ± 0.3	30.6 ± 3.8	3.9 ± 0.7	47.2 ± 2.1
		p-value (ES)	0.031* (-0.403)	0.086 (0.18)	0.03* (0.406)	0.036* (-0.223)	0.154 (0.153)	0.373 (−0.095)
	36-45	Inactive	3.3 ± 0.6	13.8 ± 4.8	1.3 ± 0.3	30.7 ± 2.7	4.3 ± 0.6	46.9 ± 2.4
		Active	3.3 ± 0.5	12.5 ± 5.1	1.3 ± 0.2	29.1 ± 3.1	4.6 ± 0.8	46.2 ± 2
		p-value (ES)	0.923 (−0.011)	0.213 (0.131)	0.914 (0.012)	0.049* (0.206)	0.26 (−0.119)	0.178 (0.14)
	46-55	Inactive	3.3 ± 0.6	13.8 ± 3.8	1.5 ± 0.3	30.5 ± 2.4	3.9 ± 0.5	46.8 ± 2.2
		Active	3.4 ± 0.3	14.6 ± 3.9	1.3 ± 0.2	31.6 ± 3.4	3.6 ± 0.4	47.9 ± 2.7
		p-value (ES)	0.022* (-0.243)	0.015* (-0.253)	< 0.001*(0.396)	0.003* (-0.318)	0.006* (0.292)	0.01* (-0.271)
	56-65	Inactive	3.5 ± 0.6	12.2 ± 5.1	1.7 ± 0.4	31.8 ± 3.4	3.6 ± 0.6	46.8 ± 2.8
		Active	3.5 ± 0.6	14.1 ± 4.5	1.4 ± 0.2	31.3 ± 2.5	3.7 ± 0.5	46.3 ± 2.6
		p-value (ES)	0.591 (0.057)	0.012* (-0.263)	< 0.001*(0.392)	0.34 (0.101)	0.388 (−0.092)	0.33 (0.103)
Males	18-25	Inactive	3.4 ± 0.5	14.4 ± 3.7	1.4 ± 0.3	30.4 ± 2.1	3.9 ± 0.4	46.5 ± 2.0
		Active	3.3 ± 0.5	14.1 ± 4.6	1.6 ± 0.3	28.5 ± 5	4.1 ± 0.9	47.2 ± 2.9
		p-value (ES)	0.147 (0.157)	0.946 (0.008)	< 0.001*(-0.631)	0.022* (0.247)	0.585 (−0.059)	0.085 (−0.185)
	26-35	Inactive	3.3 ± 0.4	14.7 ± 4.2	1.5 ± 0.3	31.1 ± 2.7	4 ± 0.6	46.7 ± 2.1
		Active	3.7 ± 0.5	13.6 ± 4.8	1.4 ± 0.3	30.8 ± 3.9	3.9 ± 0.9	47.7 ± 2.4
		p-value (ES)	< 0.001*(-0.46)	0.741 (0.036)	0.187 (0.251)	0.637 (−0.052)	0.113 (0.174)	0.017*(-0.258)
	36-45	Inactive	3.5 ± 0.3	14.9 ± 3	1.5 ± 0.3	31.7 ± 3	3.8 ± 0.8	46.7 ± 2.8
		Active	3.4 ± 0.4	15.8 ± 2.6	1.3 ± 0.2	31.2 ± 4	3.8 ± 0.6	47.4 ± 2.3
		p-value (ES)	0.847 (0.021)	0.142 (−0.155)	0.005* (0.304)	0.7 (0.042)	0.264 (−0.122)	0.263 (−0.121)
	46-55	Inactive	3.7 ± 0.5	14.4 ± 3.7	1.5 ± 0.3	31.9 ± 2.8	3.9 ± 0.8	46.7 ± 2.5
		Active	3.5 ± 0.4	14.3 ± 3.7	1.3 ± 0.3	32.2 ± 2.4	3.8 ± 0.5	47.8 ± 2.2
		p-value (ES)	0.002* (0.333)	0.59 (−0.058)	0.003* (0.576)	0.264 (−0.121)	0.767 (0.033)	0.011* (-0.276)
	56-65	Inactive	3.7 ± 0.4	13.7 ± 4.5	1.6 ± 0.3	30.1 ± 3.1	4.4 ± 0.9	46.4 ± 1.7
		Active	4 ± 0.4	16 ± 2	1.4 ± 0.3	31.7 ± 1.5	4 ± 0.5	46.9 ± 2.3
		p-value (ES)	< 0.001*(-0.46)	0.081 (−0.199)	0.001* (0.376)	0.002* (-0.351)	0.027*(0.256)	0.407 (−0.094)

* indicates results with p ≤ 0.05. ES, effect size. Results for the Student’s t-test are underlined, while results for the Mann–Whitney U test are not underlined. For the Student’s t-test, effect size is given by Cohen’s d. For the Mann–Whitney U test, effect size is given by the rank biserial correlation; Positive ES, values indicate higher values in the Active Runners group compared to Inactive Controls, while negative values indicate higher values in the Inactive Controls group. P1 – early-stance peak of the resultant knee joint reaction force; P2 – mid-stance local minimum of the resultant knee joint reaction force; P3 – late-stance peak of the resultant knee joint reaction force.

## 4 Discussion

This cross-sectional study examined differences in knee joint loading during gait according to age, sex, and physical activity using simulations in the AnyBody Modeling System. The results enabled the verification of the hypotheses, revealing significant, yet not unequivocal, relationships between the factors subjected to analysis and knee joint loading values. It was observed that older individuals were characterised by lower knee joint loading than younger individuals, but this difference was present only during selected phases of the walking gait cycle, which confirms the complex nature of the age-related differences. Differences between sexes, characterised by higher knee joint loading in men compared with that in women, were visible only in specific age groups. It was demonstrated that regular physical activity was associated with reduced loading affecting the knee joint during walking.

The following parts of the Discussion present potential biomechanical mechanisms, interpretations of the results and a discussion in the context of the existing data presented in scientific literature in the context of the research hypotheses.

### 4.1 Age-related differences in knee joint loading during walking

The results of the present study indicate that age-related differences in knee joint loading during walking are not uniform and depend on the phase of the gait cycle as well as sex and physical activity level. Although lower resultant knee joint reaction forcein older individuals was observed in selected phases of the stance, when values were normalised to body weight (BW), this pattern was not consistent across the entire gait cycle or across all subgroups. These findings highlight the complex and context-dependent nature of age-related differences in knee joint loading.

The SPM analysis (Figure 2), comparing courses of resultant knee joint reaction forces between the youngest group (18–25 years) and the oldest group (56–65 years), revealed statistically significant differences in selected phases of the walking gait cycle. Physically inactive older women were characterised by higher values at the maximum in the early stance phase–P1, whereas physically inactive younger women were characterised by higher values for P3, i.e., at the maximum in the late stance phase, indicating an age-related shift in loading towards the initial phase of contact with the ground. Such results may reflect compensatory changes in locomotor strategy resulting from a reduced capacity to generate power in the terminal gait stance phase (DeVita and Hortobagyi, 2000; Favre et al., 2014).

In the group of physically active women, the differences were less pronounced and mainly limited to the terminal part of the walking gait cycle (approximately 80%–100%), suggesting that regular running may contribute to maintaining a more stable loading profile across the lifespan. This effect may be attributable to the preservation of eccentric strength of the knee extensors and more effective control of the lower limb stabilisation in physically active individuals. Although absolute muscle force may decline with age, regular running may contribute to maintaining functional capacity under sustained loading conditions (Fukuchi and Duarte, 2019).

In physically inactive men, statistically significant differences between the oldest and youngest age groups included both the beginning (2%–9%) and the mid-part (28%–50%) of the stance phase as well as at the beginning of the swing phase. In older men, higher knee joint loading values were recorded during P3, which may indicate increased loading during the body weight transfer and compensatory engagement of the knee extensor muscles in order to maintain balance and stability (Judge et al., 1996). In turn, in physically active men, the differences were primarily concerned with the first knee loading maximum, i.e., point P1. Older participants were characterised by higher values in the initial part of the stance phase, which may result from increased stiffness of the musculotendinous structures and a reduced capacity for shock absorption (DeVita and Hortobagyi, 2000).

The analysis of extreme values (Table 2), based on single time points, confirmed these observations. In the group of physically inactive men, older participants (56–65 years) were characterised by higher values of the first extremum (P1) by approximately 0.3 BW and of the third extremum (P3) by approximately 0.5 BW in comparison with the younger group (18–25 years). In the group of physically active men, these differences were greater (P1 +0.7 BW), whereas values for P3 were comparable. Among women in the older age group, values at P1 were higher by approximately 0.2–0.3 BW. In turn, at P3, a decrease of approximately 0.6 BW was observed in physically inactive women, whereas an increase of approximately 0.2 BW was observed in physically active women.

Both the analysis of the SPM courses and the assessment of the selected extreme values (P1, P2, and P3) indicate an age-related shift in knee joint loading towards the earlier portion of the stance phase. These changes may be interpreted as a consequence of modifications in postural control strategies and impact attenuation during heel strike against the ground (Schloemer et al., 2017). Judge JO, Davis RB (Judge et al., 1996) indicate that older subjects had lower ankle plantarflexor power during the late stance phase of gait and appeared to compensate for reductions in plantarflexor power by increasing hip flexor power.

The obtained loading results are consistent with previous observations by DeVita and Hortobagyi (2000), Favre et al. (2014), Nagano and Begg (2020) as well as Buddhadev et al. (2020), who demonstrated that the effect of age on knee joint loading is context-dependent. In studies conducted under controlled walking speed conditions, lower moments and powers in older adults were observed, whereas in terms of free (self-selected) walking speeds, it was possible to more frequently observe increases in the aforesaid parameters during selected phases of gait.

Previous research indicates that age-related changes in knee joint loading during walking are characterised by (1) reduced knee extension angles and moments at heel-strike (Begg and Sparrow, 2006; Favre et al., 2014); (2) reduced knee work during stance when walking at matched speeds (DeVita and Hortobagyi, 2000); (3) redistribution of mechanical loading from distal to proximal joints (DeVita and Hortobagyi, 2000; Monaco et al., 2009) and (4) altered muscle activation patterns with increased quadriceps activity aimed at maintaining normal gait mechanics (Rudolph et al., 2007). These adaptations appear to constitute effective compensation during healthy ageing, yet they may become insufficient in the context of pathologies such as degenerative joint disease (Boyer and Andriacchi, 2016) or reduced physical activity.

### 4.2 Sex-related differences in knee joint loading during walking

Based on the SPM analysis (Figure 3), it can be observed that the hypothesis assuming higher knee joint loading in men during walking was supported only in selected subgroups. Higher knee joint loading values were observed in men in both physically active and physically inactive groups aged 56–65 years as well as in the group of physically active men aged 18–25 years. Conversely, in the 36–45-year age group, both physically active and physically inactive women exhibited significantly higher knee joint loading than men. No statistically significant sex-related differences were observed in the 26–35 and 46–55-year age groups.

These differences may arise from a different joint loading and stabilising strategy related to sex (Nicolella et al., 2012). It should be noted that the subject-related literature largely supports the hypothesis formulated in this article [e.g., Obrębska et al. (2020)], however, some studies report findings that challenge it [e.g., Loverro et al. (2019)]. Such discrepancies may result from anatomy (Nicolella et al., 2012), neuromuscular strategy (Steiner et al., 2023) and age (Inns et al., 2025) as well as physical activity.

A recurrent pattern observed across several subgroups was higher knee joint loading in men during the initial stance phase (approximately 10%–22% of the gait cycle). This phase corresponds to rapid body weight acceptance and may reflect sex-related differences in impact attenuation and lower-limb stabilisation strategies. This pattern may be associated with differences in body mass distribution, neuromuscular control, or loading acceptance strategies between sexes. From a clinical perspective, increased joint loading and excessive body mass (Nicolella et al., 2012) have been associated with a long-term risk of tissue damage or the development of degenerative changes.

### 4.3 Differences in knee joint loading associated with physical activity level

The results of the performed simulations support the hypothesis that regular engagement in physical activity may lead to adaptations of the musculoskeletal system influencing the manner in which loads are transmitted within the knee joint. Núñez-Lisboa and Dewolf (2025) demonstrated that physical activity helps maintain neuromuscular control, muscle mechanical properties and walking efficiency, while simultaneously mitigating age-related deteriorationof these functions. This may suggest that regular physical activity may contribute to maintaining a more efficient and stable locomotor pattern and, consequently, limit mechanical overload acting on knee joint structures, even under the conditions of progressive age-related changes.

Across the analysed age groups, a statistically significant association with physical activity on the resultant knee joint reaction force was observed in the youngest group of women (18–25 years) and in the 46–55 and 56–65 age groups in both sexes. Higher resultant knee joint reaction force values were recorded between 30% and 50% of the walking gait cycle in physically inactive individuals. This interval corresponds to the period of peak mechanical demand on the knee joint during the stance phase, associated with high ground reaction forces and the need to stabilise the entire lower limb. According to the literature, increased joint loading may lead to cartilage overloading and accelerate degenerative processes. Felson (2013) argues that knee osteoarthritis (OA) is largely a consequence of chronic exposure to elevated mechanical loads, leading to progressive damage of joint structures. Egloff et al. (2012) confirm that altered loading mechanisms, an increase in mechanical forces and disrupted biomechanics constitute key pathomechanical factors facilitating the development of OA. In the present study, simulations were performed in a group of healthy individuals, in whom no concerning changes were identified. The observed differences in loading levels appear to result primarily from different levels of physical activity, which, however, over the long term, may lead to an increased risk of knee joint overloading and the development of degenerative processes in individuals leading a physically inactive lifestyle. Importantly, physically inactive individuals are also frequently characterised by reduced muscle force in lower limbs (Vivan et al., 2024), which may lead to a greater reliance on passive structures (e.g., ligaments and the joint capsule) for joint stabilisation and increase the forces acting on articular cartilage (Peitola et al., 2025) and, over the long term, result in overloading and microtrauma. These findings may suggest a potentially protective association between regular physical activity and knee joint loading patterns during walking.

Notably, interesting observations are also concerned with the 12%–24% phase of the walking gait cycle, in which statistically significant differences were identified in two groups, i.e., women aged 18–25 years and men aged 56–65 years. In both cases, higher joint reaction force values were recorded in physically active individuals. This phase corresponds to the early stance phase following the heel-ground contact, during which eccentric action of the quadriceps muscle plays a key role in controlling knee joint flexion and absorbing shocks transmitted through the limb during the transmission of the bodymass (Arhos et al., 2021). Higher loading observed in physically active individuals may therefore result from a more dynamic walking pattern.

It is worth emphasising that no statistically significant differences in the impact of physical activity on knee joint loading during the stance phase were observed in the 26–35 and 36–45 age groups, in either women or men. The absence of substantial differences in participants aged 26–45 years may indicate that locomotor control and muscular stabilisation mechanisms remain relatively preserved during middle adulthood (Peitola et al., 2025), limiting the biomechanical impact of physical activity level on knee joint loading during walking.

The results obtained in the study indicate that regular physical activity may beneficially affect knee joint loading patterns during walking. In some individuals in the analysed groups, physically active ones were characterised by lower values of the resultant knee joint reaction force, which may potentially reduce the exposure of musculoskeletal structures to overloads. These findings are consistent with observations by Borgia et al. (2022), who demonstrated that regular physical activity contributes to maintaining lower limb muscle and joint function with age and helps preserve proper movement patterns. In turn, Boyer et al. (2012) provided quantitative evidence that increased physical activity may mitigate age-related deterioration in walking mechanics parameters. Although a high level of physical activity does not eliminate all ageing-related changes, it may substantially limit their magnitude and thereby support preserving musculoskeletal system functions in older adults.

In the present study, the physically active group engaged in regular running activity, which, according to the literature, may influence locomotor system function. In line with the findings of Cogliati et al. (2020), long-distance running may play an important role in mitigating age-related decline in muscle function in older individuals by improving neural impulsation to muscles. Furthermore, Hafer et al. (2019) demonstrated that middle-aged individuals who run regularly exhibit greater resistance to walking-induced fatigue of the knee extensor muscles and that intense physical activity after the age of 55 additionally protects against risk factors associated with the development of knee joint degenerative disease. It is also important that regular physical activity does not increase the risk of developing knee joint degenerative disease (Qin et al., 2018), and may even have a protective effect by slowing the progression of the disease. Overall, the present findings are consistent with previous observations suggesting that regular physical activity, including running, may be associated with more favourable knee joint loading patterns during walking and may contribute to maintaining locomotor mechanics with ageing.

### 4.4 Limitations to this work and directions for further research

One of the factors potentially affecting the obtained results is the possibility of error arising from the inaccurate placement of markers at anatomical locations. Because of the large size of the study group, this procedure was performed by more than one assessor. However, research by Malus et al. (2021) demonstrated high reliability and objectivity of anatomical marker placement, with mean coefficient values exceeding 0.8 for all segments, except the midfoot (0.683). These findings indicate that the involvement of many assessors in large cohort studies does not result in a substantial deterioration of data quality. Consequently, variability resulting from marker placement was unlikely to have had a significant impact on the simulation results.

Another limitation of the study concerns the simplifications adopted in the mathematical modelling process. The analyses involved the use of the ScalingLengthMassFat scaling method, which, based on anthropometric data (body mass and height) and fat tissue content, determines the dimensions of individual body segments and maximal muscle forces. In addition, the model was based on rigid-body assumptions and did not include subject-specific bone geometry, cartilage properties, or individual muscle architecture. Therefore, the obtained joint loading values represent computational estimates of internal musculoskeletal loading rather than direct *in vivo* measurements. Although accurate representation of muscle force capacity is particularly important in analyses requiring high mechanical loads, it should be noted that the subject of this study is walking, which does not require the generation of maximal muscle forces. For this reason, the adopted simplifications of the model should not have substantially limited the interpretation of the obtained results.

Another limitation of the study is the lack of an assessment concerning walking kinematic variability in relation to the magnitude of knee joint loading in the study group. The literature indicates that an increase in knee flexion angle during the early stance phase leads to an increase in knee joint loading (Kawaji and Kojima, 2019). At the same time, walking kinematics is largely dependent on walking speed, age, sex, and BMI (Chehab et al., 2017). In this study, which aimed to assess associations between age, sex and a physical activity level on loads, a matched-pair selection was applied. Each physically inactive participant was paired with a physically active individual characterised by a comparable walking speed and BMI values, differing primarily in the level of physical activity. Such an approach aimed to minimise the effect of potential confounding factors.

The concepts presented and the methods applied in the study provide potential for further development. In the future research, it would be recommendable to take into account more detailed, individual data concerning body composition, including the regional distribution of fat tissue and muscle mass in specific segments. It would be advisable to extend analyses to include a comprehensive assessment of walking kinematics. This would allow even more precise identification of the mechanisms through which physical activity, age and sex affect the locomotor system loading during walking.

## 5 Conclusion

The results of the study demonstrated that age, sex and a physical activity level have a significant connection with internal knee joint loading during walking. Older individuals generally exhibited lower knee joint loading compared with younger individuals, when expressed relative to body weight (BW), although those differences were phase-dependent and not consistent across all analysed subgroups. In turn, men tended to exhibit higher internal joint forces than women, particularly in selected age groups. Regular physical activity was associated with a reduction in internal loading, which may suggest a potentially protective association with knee joint biomechanics during walking.

The results of the study emphasize the importance of both individual demographic features and lifestyle in shaping knee joint biomechanics, which may have significant implications in the prevention of degenerative changes and the design of training programmes.

## Data Availability

The raw data supporting the conclusions of this article will be made available by the authors, without undue reservation.
