# Supplementary material for: Age, sex and physical activity differences in the knee joint loadings during walking—a cross-sectional study
Source: Front Bioeng Biotechnol. 2026 Jun 24;14:1850746. doi: 10.3389/fbioe.2026.1850746 (PMC13341928; doi:10.3389/fbioe.2026.1850746)
Supplement: Supplementary file 1 [file Supplementaryfile1.docx]

**SUPPLEMENTARY INFORMATION**

|  |  | **Age** | |
| --- | --- | --- | --- |
|  |  | **Inactive controls** | **Active runners** |
| **Sex** | **Female** | 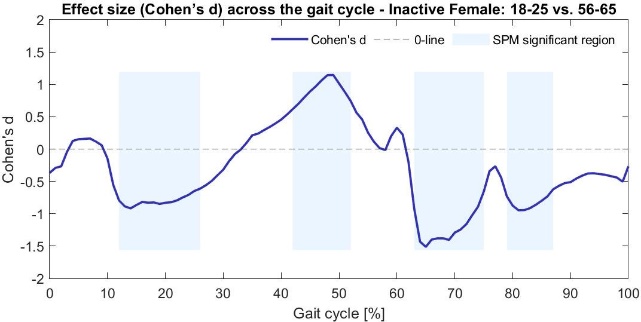 | 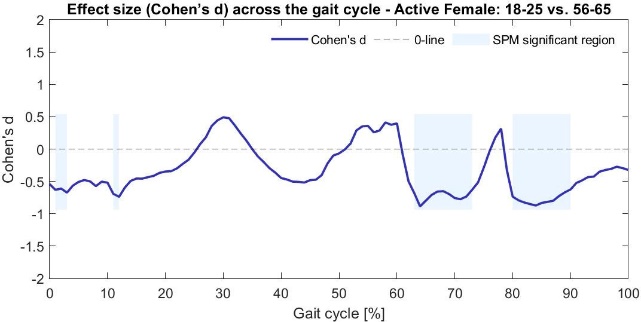 |
|  | **Male** | 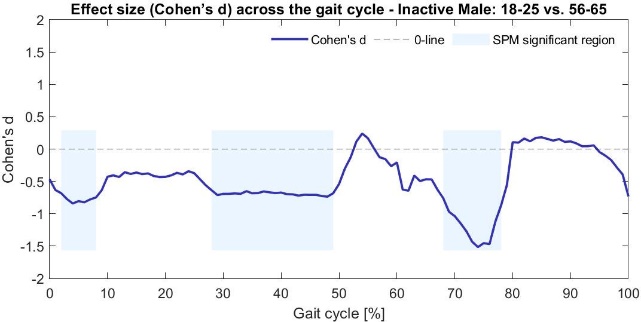 | 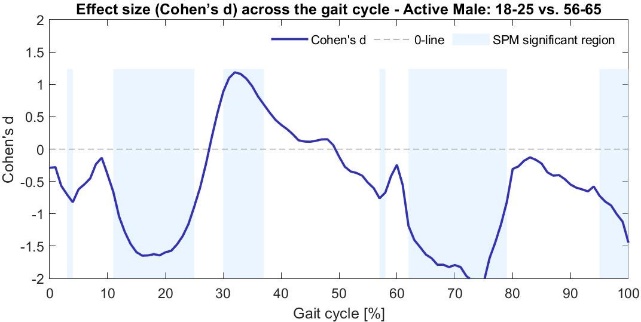 |

Supplement 1. Cohen’s d values for comparisons of resultant knee joint reaction forces according to age (18–25 vs. 56–65 years). The supplement presents average Cohen’s d values for walking gait cycle intervals (0–100%) in which statistically significant differences were identified (SPM{t}, p ≤ 0.05).

|  |  | **Physical activity** | |
| --- | --- | --- | --- |
|  |  | **Inactive controls** | **Active runners** |
| **Age groups** | **18-25** | 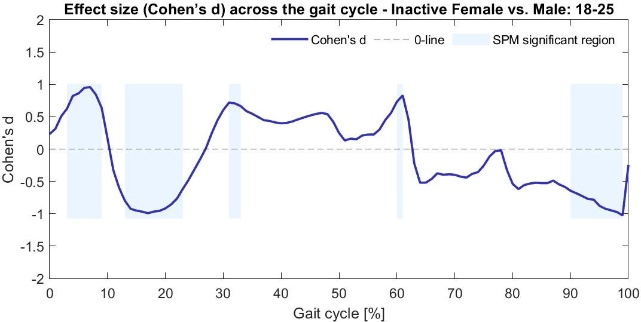 | 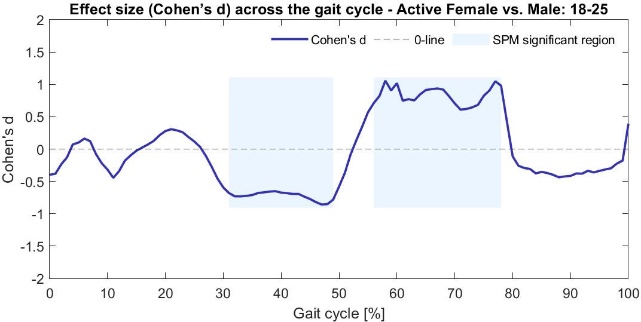 |
|  | **26-35** | 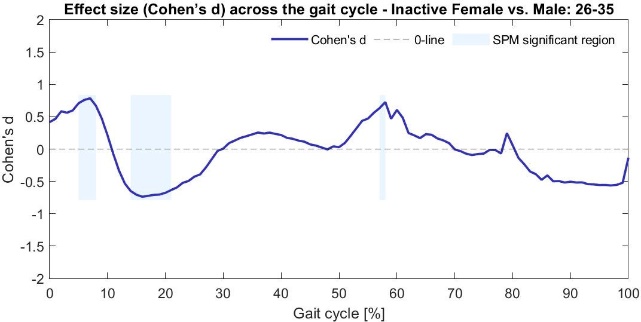 | 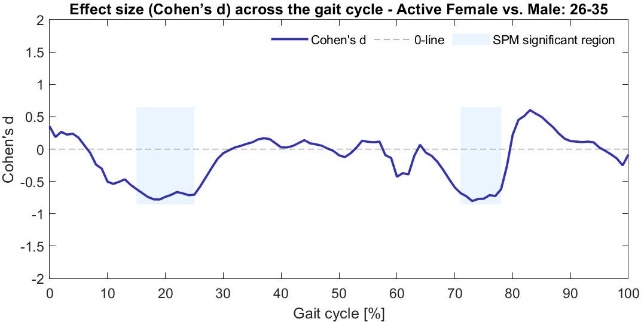 |
|  | **36-45** | **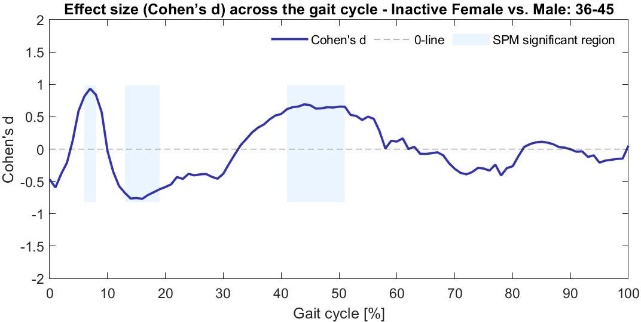** | 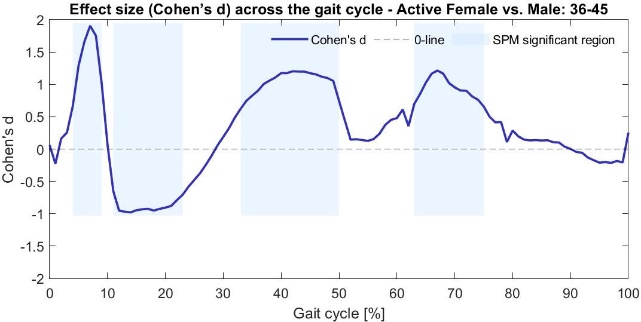 |
|  | **46-55** | **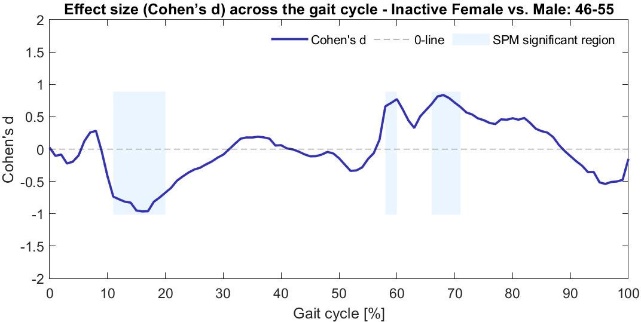** | 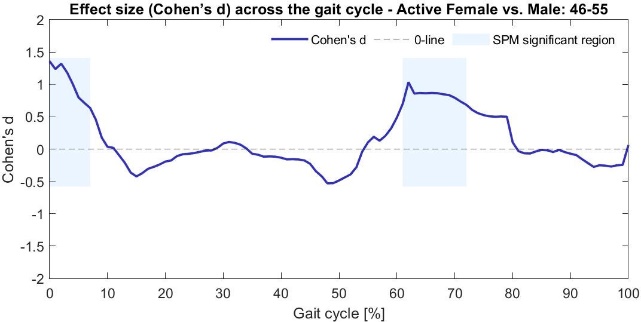 |
|  | **56-65** | 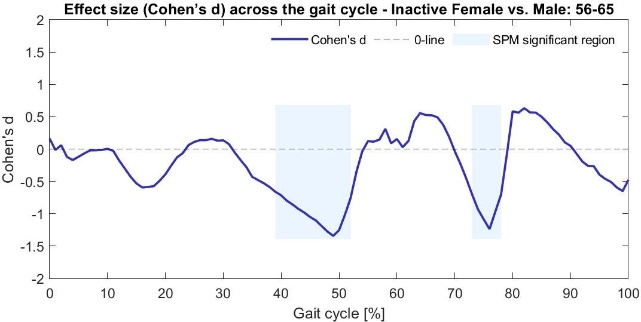 | 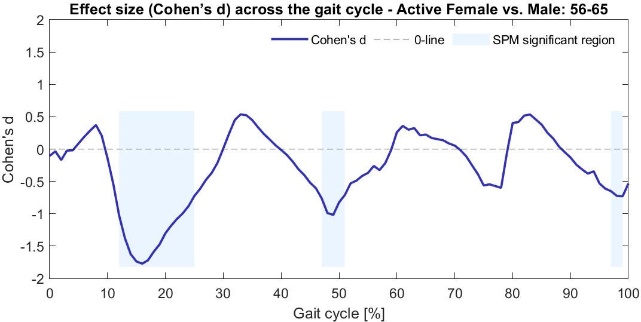 |

Supplement 2. Cohen’s d values for comparisons of resultant knee joint reaction forces according to sex (women vs. men). The diagram presents average Cohen’s d values for walking gait cycle intervals (0–100%) in which statistically significant differences were identified (SPM{t}, p ≤ 0.05).

|  |  | **Sex** | |
| --- | --- | --- | --- |
|  |  | **Female** | **Male** |
| **Age groups** | **18-25** | 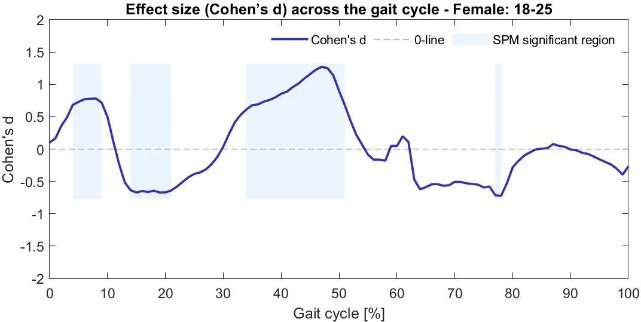 | 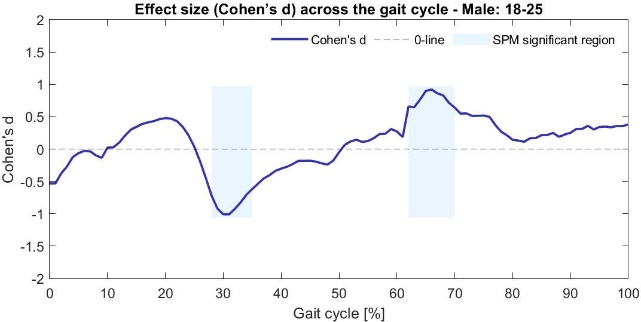 |
|  | **26-35** | **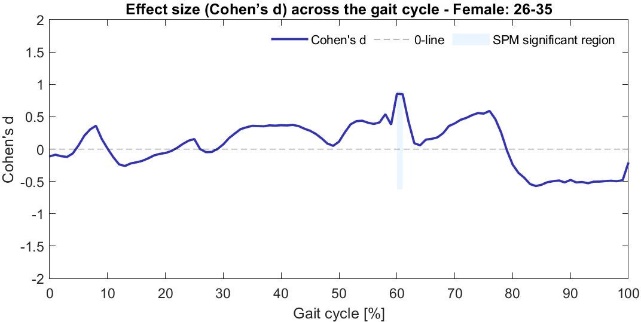** | **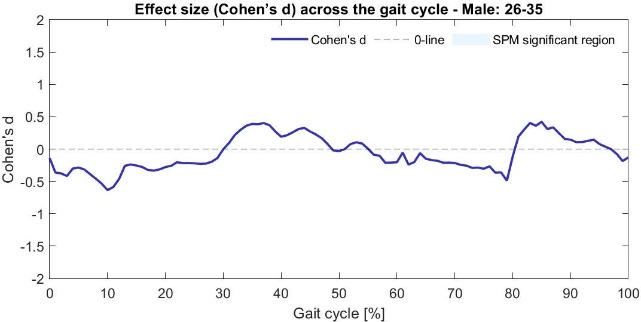** |
|  | **36-45** | **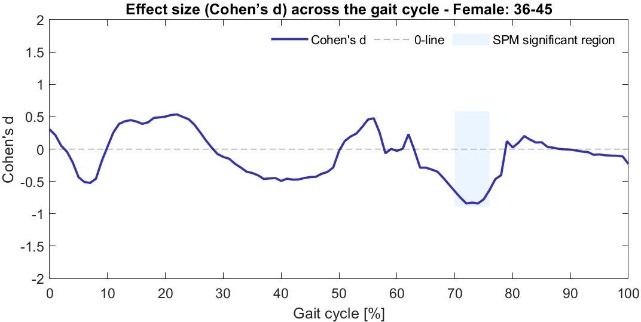** | **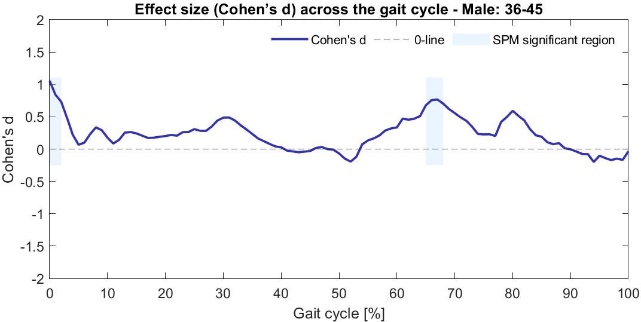** |
|  | **46-55** | 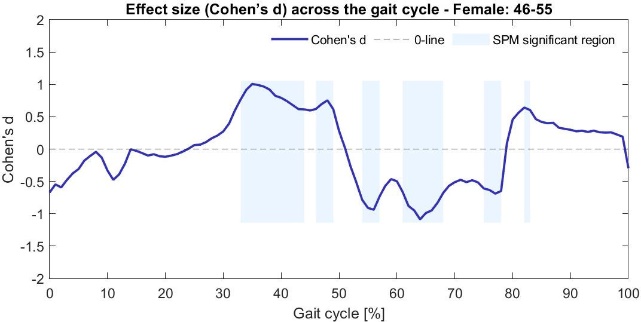 | **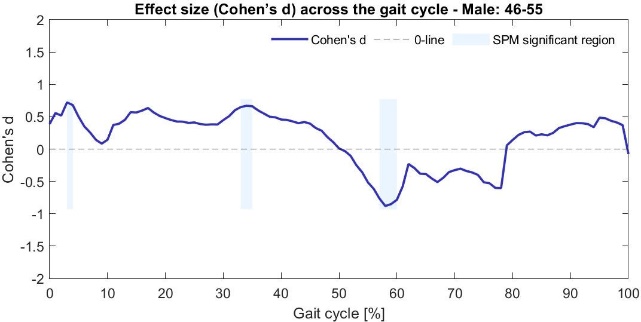** |
|  | **56-65** | **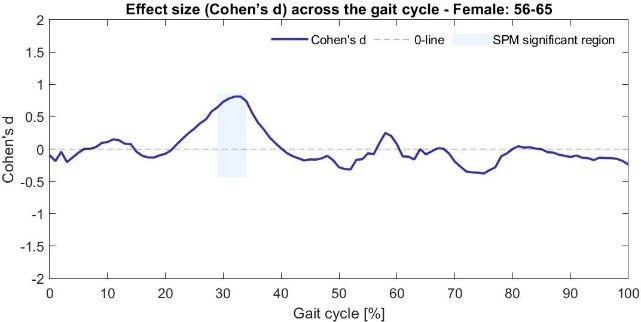** | **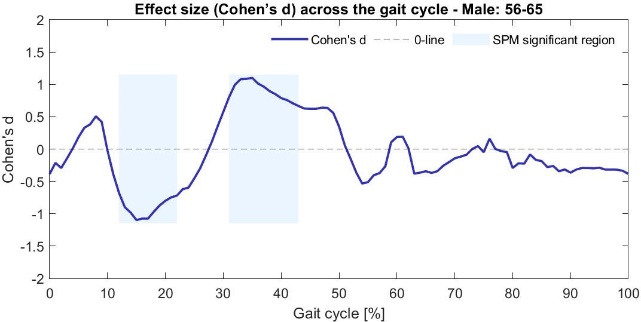** |

Supplement 3. Cohen’s d values for comparisons of resultant knee joint reaction forces according to physical activity level (active vs. inactive individuals). The supplement presents average Cohen’s d values for walking gait cycle intervals (0–100%) in which statistically significant differences were identified (SPM{t}, p ≤ 0.05).
